# Do bullae and emphysema increase risk of pneumothorax in silicosis?

**DOI:** 10.1186/1745-6673-2-8

**Published:** 2007-09-15

**Authors:** Iraj Mohebbi, Ebrahim Hassani, Shaker Salarilak, Abdul Rahman Bahrami

**Affiliations:** 1 Department of Occupational Medicine, Urmia University of Medical Sciences, Urmia, Iran; 2 Department of Anesthesiology, Urmia University of Medical Sciences, Urmia, Iran; 3 Department of Community Medicine, Urmia University of Medical Sciences, Urmia, Iran; 4 Department of Occupational Health, Hamadan University of Medical Sciences, Hamadan, Iran

## Abstract

**Background:**

The occurrence of occupational lung diseases is decreasing due to improvements in occupational health in recent years; however, silicosis and its complications remain important occupational health problems. We have studied the role of emphysema and bullae as predictive factors of secondary spontaneous pneumothorax in acute and accelerated silicosis.

**Methods:**

This study was carried out using questionnaire items on occupational history and conventional computed tomography of lungs. Differences between two groups (silicosis with and without secondary spontaneous pneumothorax) in terms of age, interval of exposure-diagnosis and therefore silica exposure duration were assessed by independent *t*-test. Fisher's exact test was used to determine the association between secondary spontaneous pneumothorax and both emphysema and bullae.

**Results:**

We found a significant association between secondary spontaneous pneumothorax and bullae in acute and accelerated silicosis.

**Conclusion:**

Pneumothorax in silicosis could be attributed to previous bullae.

## Background

Pneumothorax is the presence of air in the pleural cavity [1]. The spontaneous form is generally due to the rupture of subpleural blebs. Clinical diagnosis of pneumothorax is established by history, physical examination and where possible, by radiological investigations [2-4]. Secondary spontaneous pneumothorax (SSP) occurs as a complication of an underlying lung disease, which can be identified. Chronic obstructive pulmonary disease (COPD) and *Pneumocystis carinii *pneumonia are the most common conditions associated with SSP [1,5]. Pleural involvement in silicosis is rare and SSP is the only recognised pleural complication of silicosis [6-11]. SSP is usually unilateral, when it occurs in the course of silicosis. There are only a few reports in which patients with silicosis had bilateral SSP [9,11]. Some investigators have reported that emphysema and bullae formation may lead to the occurrence of SSP [3,12]. Emphysema is defined as enlargement of airspaces distal to the terminal bronchiole, accompanied by destructive changes of alveolar walls [13]. It is usually classified into the following three main subtypes: centrilobular emphysema; panlobular emphysema; and paraseptal emphysema [14]. Bullae can develop in association with any type of emphysema. A bulla is a sharply demarcated area with a diameter greater than 1 cm and possessing a wall less than 1 mm in thickness [15].

This case-control study was performed to evaluate the association between SSP and both emphysema and bullae on the basis of lung conventional computerized tomography (CCT) scan in the non-smoker silicotic patients.

## Methods

We studied all of registered silicotic patients who had immigrated as seasonal temporary workers from West Azarbaijan province to the stone grinding factories of Azandarian area. Pneumothorax was defined by detection of the thin, visceral line in the expiratory postero-anterior chest radiographs that was displaced from the chest wall. Those who suffered pneumothorax selected as case group and others as control.

### Characteristics of the factories and study population

From September 2000 to March 2006, 21 subjects with silicosis had been registered in the Urmia Occupational Medical Center of West Azarbaijan province in Iran. They had immigrated as seasonal workers from West Azarbaijan province (in the west of Iran) to the Azandarian area, a suburb of Hamadan province in the center of Iran. This area located 750 Km far away from West Azarbaijan province, and also is a base for stone-cutting and stone-grinding factories in Hamadan province. Each factory usually employs 5 to 10 seasonal temporary workers from various districts. We studied only workers who were resident in the West Azarbaijan province with history of employment in aforementioned factories. We did not have accesses to other workers, because they were scattered in other provinces. All of the workers who are included in this study had previously worked in the same stone-grinding factories in the Azandarian area. They did not have any reported silica exposure before working at those workplaces. They described the working environment as being very dusty, with no provision of engineering control or exhaust ventilation.

### Diagnostic methods

Diagnosis of silicosis was made on the basis of clinical findings, chest radiological criteria in accordance with the ILO International Classification of Radiographs of Pneumoconiosis, an unequivocal history of substantial silica dust exposure and an appropriate interval of time after exposure. We defined acute and accelerated silicosis according to NIOSH definition [16]. Patients with silicosis were entered into the study if the high voltage posterior-anterior radiograph of the chest with acceptable quality showed reticular-nodular shadowing at least 1/1 profusion grade according to the ILO system. Expiratory postero-anterior chest radiography was reviewed for detection of pneumothorax and also SSP was defined by identification of the thin, visceral line that was displaced from the chest wall. In each subject, CCT of lung was reviewed for recognition of emphysema and bullae. For each subject, a detailed history of work occupations was recorded as follows: (a) the age of onset of exposure to silica dust; (b) the age when the exposure to silica dust ended; (c) the age when the initial diagnosis was made.

### CCT procedures

The scanners of GE 4000 SYTEC (GE Medical Systems, Milwaukee, WI, USA) and X VISION-EX CT (Toshiba; Tokyo, Japan) were used for recognition of emphysema and bullae. For each subject, CCT scans had been obtained with 10 mm collimation at 1 cm intervals from the upper to the lower thorax in the supine position. Emphysema was defined as well-delineated focal peripheral areas surrounded by a thin wall and less than 10 mm in diameter. If an area of emphysema was greater than 1 cm in diameter, this was defined as a bulla.

### Stone grinding process

There was the same process in the 20 stone grinding factories as follows: The quartz stone is first put into a jaw crusher where large stone is broken into smaller pieces, which are then taken through a conveyor belt to disintegrator, which makes powder out of these small pieces. It is then separated according to its fineness through a vibrating screen. The quartz powder is also passed through a magnetic separator in order to remove the extraneous ferrous material from the product raw material.

### Characteristics of the occupational exposure

In the Azandarian area where the patients had been employed, all of the stone-grinding factories were selected for the assessment of environmental exposure of silica particles. The determination of quartz was carried out according to the NIOSH method number 7500. A rotameter was used to adjust the flow. The respirable dust samples were collected on 25 mm cellulose acetate filters (pore size 0.8 μm) placed in a 25 mm conductive plastic cyclone. The cyclone was attached to the worker's overalls as closely as possible to the face in order to determine respirable dust in the breathing zone. The filters were conditioned in desiccator environmental chamber for 24 hours at 25°C and weighed before and after testing to determine total penetrating weights. The analysis was done by X-ray diffraction (XRD) using a Siemens Model D5000 diffractometer equipped with variable slit in research laboratory of X-ray at the Faculty of Science, Tehran University.

### Statistical analysis

Statistical analysis was performed using commercially available software (SPSS 10.0 for Windows; SPSS; Chicago, IL, USA). Differences between the two groups (silicosis with SSP and silicosis without SSP) in age, interval between the onset of exposure to silica dust and clinical diagnosis of silicosis, and silica exposure duration were assessed by independent *t*-test. We used Fisher's exact test to determine the association between SSP and predictive factors that included emphysema and bullae.

## Results

The total working period was 12 to 14 h a day for 1 to 5 consecutive years. All of the subjects were male and non-smokers with the youngest being 20 and the oldest 79, of whom 18 (86%) were younger than 40. The acute and accelerated silicosis were found in 43% and 57%, respectively. Latency periods of the acute and accelerated forms were 3.2 ± 0.83 (mean ± SD) and 6.4 ± 1.6 (mean ± SD) years, respectively. In the chest radiographs, 3(14.3%) had radiological profusion category 1, and8(38.1%) category 2. 10 (47.7%) showed category3. The most common subcategories of small opacities profusion was 3/3 (42.9%).

In patients who experienced SSP, the interval between the onset of exposure to silica dust and clinical diagnosis of silicosis was 3.2 ± 1.1 (mean ± SD) years and in those without SSP, it was 5.42 ± 1.60 years. The mean of exposure duration in patients with SSP was 2.14 ± 1 years and in those without SSP, it was 2.86 ± 1.2 years. The difference in age between the two groups at the end of exposure was not significant. There was a significant difference between the two groups in the interval from first silica exposure to diagnosis of SSP and no significant differences in exposure duration (Table 1).

**Table 1:** Comparison of age, interval between onset of exposure and clinical diagnosis, and exposure duration between the two groups

Variable	Silicosis with SSP(mean ± SD)	Silicosis without SSP (mean ± SD)	T	p value
Age at the end of exposure (years)	26.43 ± 5.85	34.50 ± 17.7	1.22	0.073
Interval between the onset of exposure and clinical diagnosis (years)	3.2 ± 1.1	5.42 ± 1.6	3.5	0.002
Exposure duration (years)	2.14 ± 1	2.86 ± 1.2	1.3	0.19

**Figure 1:**
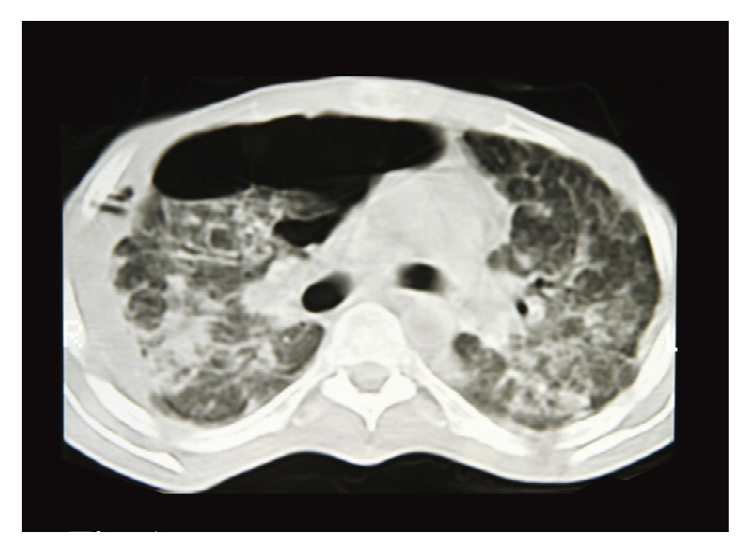
CT. 1: Large localized pneumothorax in right lung. 2: Mixed alveolar and interstitial fibrosis. 3: Pleural thickening in right lung. 4: Several bullae in right lung. 5: Alveolar and interstitial shadowing. 6: Paraseptal emphysema in anterior segment of left upper lobe.

**Figure 2:**
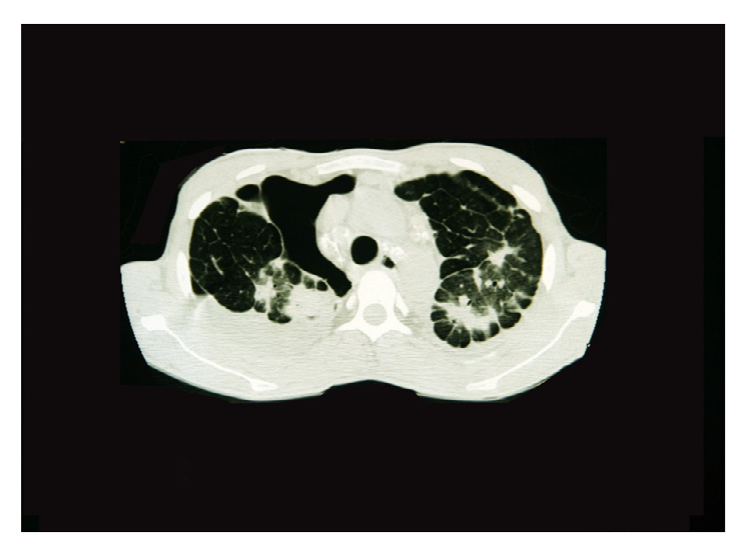
CT. 1: Hydropneumothorax in right lung. 2: Multiple bullae in both lungs. 3: Paraseptal emphysema in left lung. 4: Mixed alveolar and interstitial pattern as silicoproteinosis and fibrosis. 5: Air bronchogram

In our study, SSP was identified in 34% of subjects (Figure 1) of which 19% was bilateral. Emphysema and bullae were found in 49% and 52%, respectively (Figure 2). There was a high odds ratio between SSP and emphysema (odds ratio = 10.8 CI; 0.997–117). We found that the probabilities of SSP were higher in those with bullae (odds ratio = 15 CI; 1.3–168), as shown in Table 2.

**Table 2:** Odds ratio and comparison of risk factors between the two groups

Risk Factor		Pneumothorax		Odds ratio	95% Confidence Interval
				
		No	Yes		
Bullae	No	10	1	15	1.3–168
	Yes	4	6		

Emphysema	No	9	1	10.8	.997–117
	Yes	5	6		

The silica particles in the ambient air at all of the factories were higher than OSHA level, as shown in Table 3.

**Table 3:** Exposure assessment in each industrial unit (mg/m 3)

Industrial unit	Total dust	Total Respirable dust	Respirable quartz
1	1355.37	66.70	17.35
2	1438.21	78.34	19.60
3	1442.21	79.45	17.89
4	1472.73	83.87	20.34
5	1542.32	88.45	22.87
6	1618.71	94.45	26.00
7	1624.45	83.45	23.90
8	1641.34	105.54	31.89
9	1645.13	104.79	32.90
10	1725.75	109.86	31.00
11	1747.00	111.27	39.90
12	1747.00	121.32	42.27
13	1747.45	121.30	42.80
14	1836.73	126.97	44.90
15	1845.37	127.12	44.89
16	1924.14	134.12	45.89
17	1995.34	134.78	50.90
18	2032.80	159.45	56.90
19	2058.81	141.00	48.50
20	2067.83	134.60	44.80

## Discussion

The results of the present study demonstrate that in individuals with advanced silicosis, SSP is significantly associated with the presence of bullae. The results also indicate that advanced silicosis causes distal airspace enlargement independent of smoking.

To the best of our knowledge, predictive factors of SSP as a complication of silicosis have not been extensively described in the literature [1,5]. Choi et al. studied 458 patients who underwent transthoracic needle biopsy (TTNB). They found a significantly higher (p < 0.001) risk of pneumothorax in patients with emphysema and concluded that development of pneumothorax may be prevented by the elastic recoil of the normal lung parenchyma and pleura [17]. Mitlehner et al. suggested that the presence of bullae in patients with primary spontaneous pneumothorax has no predictive value for the future development of recurrences [18]. Conversely, bullae rupture in SSP appears to be due to local airway obstruction, emphysema susceptibility, and the presence of bronchial abnormalities [3]. Functionally, massive fibrosis results in stiff nondistensible lungs with increased elastic recoil [19]. In advanced silicosis, coalescence of perinodular emphysematous regions may lead to formation of macroscopic blebs, which can rupture causing a pneumothorax [12]. Our findings were consistent with these findings and indicate that the occurrence of SSP could be attributed to the presence of bullae.

In the absence of smoking, coal pneumoconiosis and confluent silicosis are associated with emphysematous changes in the lungs [20]. Some investigators have reported that emphysema with silicosis has also been observed to occur independently of smoking [21,22]. Emphysema is common in silicosis and has been attributed as the major cause of corpulmonale and disability rather than fibrosis by some investigators [23].

In this study, all of the subjects were non-smokers and the results confirmed that emphysematous changes are common in non-smoker silicotic patients.

In a case series study, Kawano et al. suggested that there was no correlation between the onset of SSP and duration of occupational exposure to silica [24]. Our findings support this hypothesis as shown in Table 1. According to an investigation by Bahrami and Mahjub in the stone-grinding factories where the study subjects had been employed, the concentration of silica compounds in the ambient air had been 25–50 times higher than Occupational Safety and Health Administration (OSHA) levels [25]. Our findings were consistent with these findings. Our study also showed that the interval between the onset of exposure and clinical diagnosis of silicosis was statistically different for both groups, but both of them were exposed to extremely high levels of respirable silica. In summary, development of SSP may be enhanced by increased elastic recoil of the lung parenchyma, and bullae rupture. There is a high probability of SSP occurring in acute and accelerated silicosis.

Historically, people employed in stone-grinding factories are from lower income backgrounds and have not had the advantage of regular medical surveillance or specialized care until their conditions become very advanced. A significant difference between the two groups in the interval from first silica dust exposure to clinical diagnosis of silicosis might possibly be a confounding issue that silicosis may be misdiagnosed due to lack of appropriate health surveillance. We concluded that a brief, but intensive exposure to silica dust could cause SSP after a short latency period. It is recommended that an effective hygiene program be implemented to monitor the health of these workers.

Our methodology for assessing odds ratios and the association between pneumothorax and both bullae and emphysema in silicosis has both strengths and weaknesses. Its strengths are as follows. All subjects had worked in the same unregulated stone-grinding workplaces and had no history of smoking, underlying disease of COPD and/or other mineral dust exposure. Information was available on the specific occupational exposure to silica powder in the stone-grinding factories. Although the sample size in our study was only 21, to the best of our knowledge, it is larger than any previous study of pneumothorax in silicosis worldwide, so its major strength is due to the number of cases. A final strength is that information was available on possible confounding factors such as tobacco use.

The main limitation of our study also concerns the number of subjects; however, we attempted to find all of scattered workers. We used the opportunity and discussed the implications for enforcement of regulations on a national level to prevent the occurrence of silicosis (especially in Hamadan province where this outbreak had occurred). Due to the rarity of acute and accelerated silicosis and also SSP in silicosis, statistical analysis of binary outcomes is almost always based on odds ratios and it is the same as the risk ratio [26]. Therefore, we believe this study might be a good estimation of odds ratio or relative risk of SSP in acute and accelerated silicosis.

## Conclusion

Our study findings reemphasize the clinical importance of SSP and its association with bullae. The results also indicate that advanced silicosis causes distal airspace enlargement independently of smoking.

## Abbreviations

CCT: Conventional computerized tomography

COPD: Chronic obstructive pulmonary disease

ILO: International Labour Office

NIOSH: National Institute for Occupational Safety and Health

OSHA: Occupational Safety and Health Administration

SSP: Secondary spontaneous pneumothorax

TTNB: Transthoracic needle biopsy

## Competing interests

We have not received any financial support or grant from any organization for carrying out this research, and all of expenditure has been met by the researchers with the aim of benefiting humanity. The authors declare that they have no completing interests.

## Authors' contributions

Iraj Mohebbi carried out the clinical and imaging studies, participated in the study design, sequence alignment and drafted the manuscript. Ebrahim Hassani carried out the scientific editing of the manuscript. Shaker Salarilak performed the statistical data analysis/interpretation. Abdul Rahman Bahrami helped in the assessment of environmental monitoring. All authors read and approved the final manuscript.
